# Various Presentations of Scrub Typhus: A Case Series

**DOI:** 10.7759/cureus.64981

**Published:** 2024-07-20

**Authors:** Vasu Saini, Sana Irfan Khan, Anita Vincent, Nutan Singh, Shafeeque Kuniabdullah

**Affiliations:** 1 Pediatrics, Shri Guru Ram Rai Institute of Medical and Health Science, Dehradun, IND; 2 Pediatrics, SUNY Downstate Medical Center, New York, USA; 3 Internal Medicine, Karnataka Institute of Medical Sciences, Hubli, IND; 4 Pediatrics, Doon Medical College, Dehradun, IND; 5 Pediatrics, Boston Children's Hospital, Boston, USA

**Keywords:** inguinal lymphadenopathy, fever with jaundice, gross hematuria, meningo-encephalitis, scrub typhus

## Abstract

Scrub typhus is caused by *Orientia tsutsugamushi*, a Gram-negative coccobacillus. It comprises three strains: Karp, Gilliam, and Kato. Cases of scrub typhus are usually found in the Asia-Pacific region, and their presentation may range from minimal symptoms to multi-organ involvement, with or without the presence of an eschar mark. Varying manifestations of scrub typhus, such as gangrene, meningoencephalitis, anemia with jaundice, and hematuria, have been observed. In the Kumaun region of northern India, there has been a surge in the number of scrub typhus cases. Typically, this disease is accompanied by an eschar mark, but occasionally it can manifest without one. We report a series of four cases presenting with various unusual symptoms such as gangrene of the limbs, meningoencephalitis, jaundice, and hematuria. Serology for scrub typhus should be considered in all patients with acute febrile illness not responding to treatment, especially in mountainous regions, to prevent the associated mortality.

## Introduction

*Orientia tsutsugamushi* is a Gram-negative coccobacillus with three strains: Karp, Gillium, and Kato. It is the causative agent of scrub typhus. Immunity against one strain of infection does not provide immunity against another strain of infection. Scrub typhus is endemic in the Asia-Pacific region, including countries such as India, China, Korea, Pakistan, Taiwan, Japan, Thailand, and Malaysia, as well as northern Australia. Chiggers, also known as larval trombiculid mites, serve as carriers of scrub typhus [1]. The illness may manifest with symptoms including headache, anorexia, malaise, and fever after a 7-10-day period of incubation, followed by rashes and eschar. The infection can present with a range of symptoms, varying from minimal signs to multi-organ failure. A lethal infection was observed in approximately 4% of hospitalized patients [2,3].

Severe sickness and consequences associated with this condition are more common in older people. Some patients have a localized eschar, or necrotic skin lesion, at the location of the infected chigger bite. Respiratory symptoms, significant kidney impairment, and generalized lymphadenopathy are also prevalent. Acute respiratory distress syndrome (ARDS) may develop in rare cases [4]. Meningitis or encephalitis also may result from blood vessel involvement in the central nervous system (CNS). Scrub typhus infection may present with minimal symptoms or involve multiple organs, with or without the presence of an eschar mark.

## Case presentation

Case I

**Figure 1:**
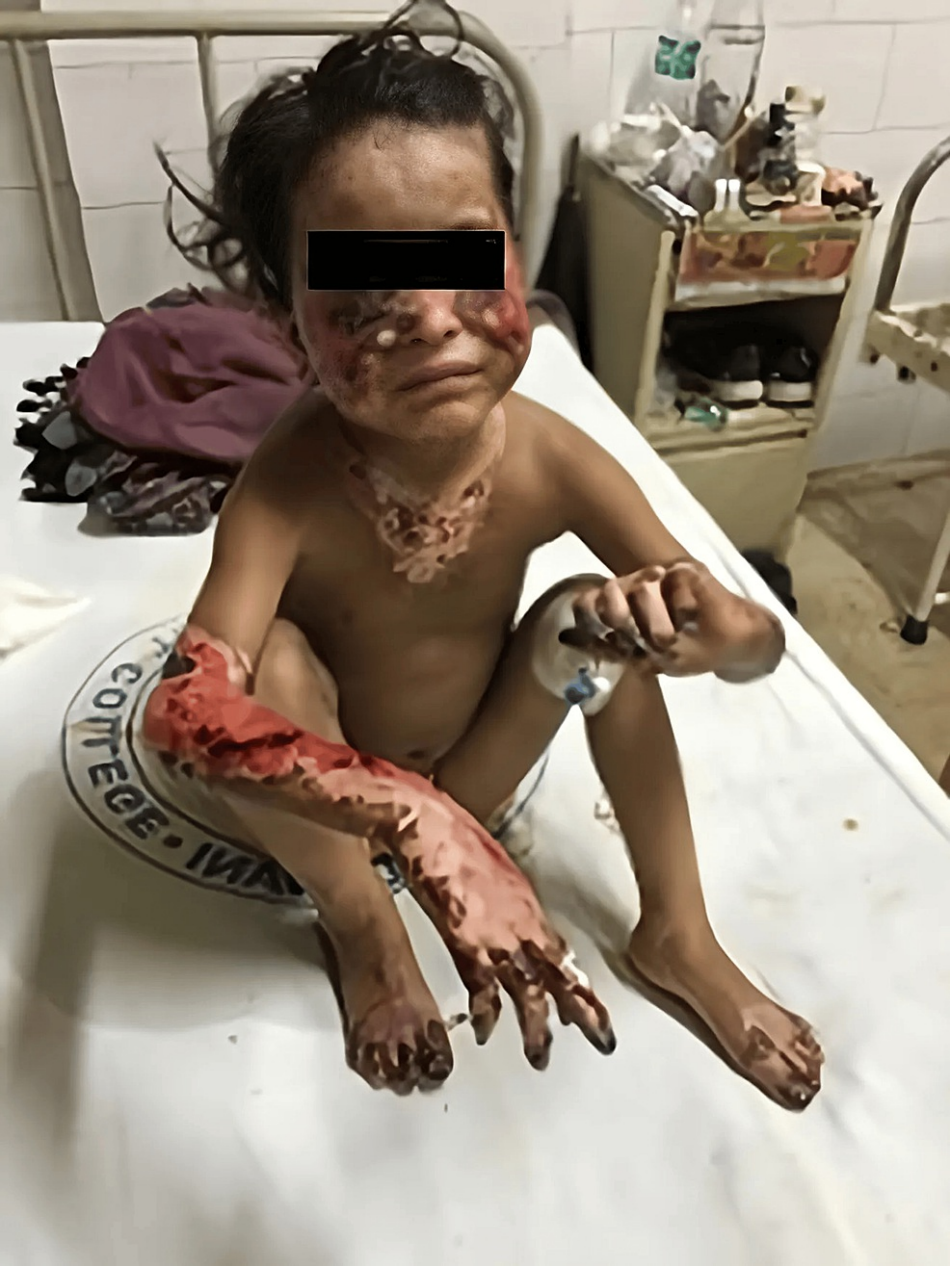
An eight-year-old female child with blackening of the bilateral forearm, hands, feet, face, and upper trunk

The district hospital in Pithoragarh referred an 8-year-old female child with complaints of blackening of the bilateral forearm, hands, feet, face, and upper trunk for one day to the department of surgery at GMC Haldwani (Figure 1). After assessment, the patient was sent to the pediatric department for a second opinion. According to the patient's father, the condition had started nine days ago with an itching in the right forearm while the child had been engaged in field sports. It had escalated to swelling across the bilateral forearms and face two hours after the itching began. After four to five days, there had been numerous blisters all over the forearms and face, and then the forearms, hands, feet, face, and upper trunk had turned black. There were no prior instances of rash, bleeding from any place, joint pain, jaundice, or hematuria.

On examining the patient, her heart rate was 102 beats per minute, respiratory rate was 18 breaths per minute, blood pressure was 108/70 mmHg, and temperature was 101 °F. Bilateral submandibular and inguinal lymphadenopathy were noted, along with gangrene over the malar region of the face, upper trunk, forearms, and digits of the hands and feet. All peripheral pulses were normal. All infectious causes of fever, such as dengue, malaria, chikungunya, and typhoid, were negative. Hepatitis B surface antigen (HBsAg), hepatitis C virus (HCV), human immunodeficiency virus (HIV), antinuclear antibody (ANA), and double-stranded DNA (dsDNA) were also negative (to rule out other causes of vasculitis). Color Doppler findings for all four limbs were normal. The 2D echocardiogram was also normal. After a positive scrub serology, doxycycline at 4 mg/kg per day in two divided doses (for five days), aspirin at 5 mg/kg per day orally, and pentoxifylline at 20 mg/kg per day orally in three divided doses (for seven days), along with further supportive care, were administered as a supplemental treatment. On follow-up after seven days, the gangrenous lesions had disappeared, and the patient became asymptomatic.

Case II

A 12-year-old male presented to GMC Haldwani with a fever persisting for eight days, a headache for seven days, abnormal body movements for two days, and altered sensorium for two days. There was no history of convulsions, jaundice, joint pain, bleeding manifestations, or abdominal pain. The patient's heart rate was 110 beats per minute, respiratory rate was 22 breaths per minute, blood pressure was 110/72 mmHg, and temperature was 101.2 °F. The Glasgow Coma Scale score was E2V1M3, and meningeal signs were present throughout the examination. However, the rest of the systemic examination yielded normal results. He was put on a ventilator due to poor general condition.

Despite receiving treatment (supportive and antiepileptic), his condition did not improve. Scrub typhus serology returned positive, and hence intravenous azithromycin at 10 mg/kg per day single dose was started and given for five days, to which the patient responded well. His sensorium improved, and weaning from the ventilator was successful. A follow-up exam after two weeks showed a normal electroencephalogram (EEG), and no routine antiepileptics were given. CT and lumbar puncture (LP) were not performed due to the patient's poor general condition. All other infectious causes of fever were ruled out, similar to Case I.

Case III

**Figure 2:**
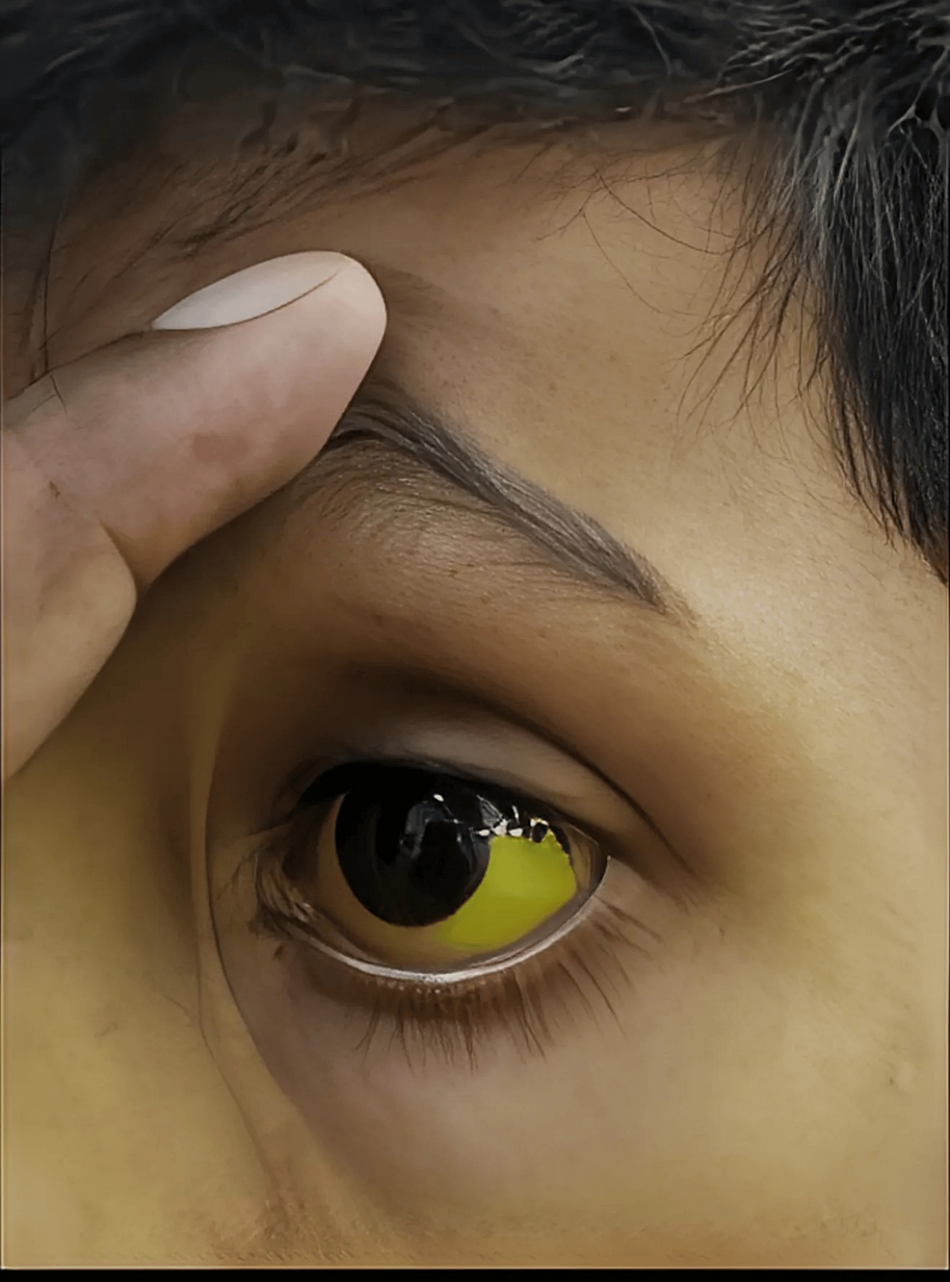
Patient exhibiting scleral icterus

A 10-year-old boy presented to GMC Haldwani complaining of fever for five days, yellowing of the eyes (Figure 2) for five days, breathing difficulty for two days, and abdominal pain for two days. There was no history of joint pain, rashes, bleeding manifestations, headaches, or irregular sleep patterns. Upon evaluation, there were pallor and icterus present but no lymphadenopathy or edema. The patient had a temperature of 102 °F, a respiratory rate of 48 breaths per minute, blood pressure of 106/70 mmHg, and a heart rate of 120 beats per minute.

The following findings were noted: CNS - within normal limits; respiratory - tachypnea, nasal flaring, and subcostal retractions with bilateral equal air entry; abdomen - soft with tenderness in the right hypochondrium, liver span measuring 13 cm; cardiovascular - S1 and S2 present, with a hemic murmur noted across the lower left sternal border. All infectious causes of fever were ruled out as in Case I. The general blood picture showed microcytic hypochromic anemia. Additionally, HBsAg, HCV, and hepatitis A tests were negative, and APTT and INR were normal. An ultrasound of the whole abdomen suggested hepatomegaly. Scrub immunoglobulin M (IgM) serology came out positive, and the patient was treated with doxycycline at 4 mg/kg per day 12 hourly for five days. Packed red blood cells (pRBCs) were transfused. The patient responded well to the treatment and became asymptomatic after five days. Follow-up liver function tests came back normal after five days, and oral iron was started for anemia.

Case IV

A 13-year-old boy was brought to GMC Haldwani from the district hospital with complaints of fever for 10 days, rashes over the body for five days, and red discoloration of urine for two days. There was no previous history of sore throat, abdominal pain, frequent urination, joint pain, or other bleeding symptoms. Upon examination, the patient had a temperature of 101.8 °F, a respiratory rate of 22 breaths per minute, a heart rate of 110 beats per minute, and a blood pressure of 110/80 mmHg. General examination revealed no pallor, icterus, edema, or lymphadenopathy. The CNS examination was within normal limits, whereas the respiratory system revealed tachypnea. Local examination identified non-blanchable petechial rashes over the abdomen and on both the upper and lower limbs.

Initially, the most likely provisional diagnosis, in this case, was acute glomerulonephritis/ dengue; however, the blood pressure was normal, complement levels were normal, and other infectious causes were also negative. Then, scrub typhus serology was sent and came back positive. Hematuria and other symptoms disappeared after three days of treatment. The patient was discharged after five days and did not return for a follow-up examination. Urine examination showed no RBC casts, with RBCs numbering 50-55 per high power field (HPF), and no WBCs, bacteria, or pus cells were detected.

Doxycycline (4 mg/kg per day twice a day) was given to all patients except Case 2, and all patients responded well to the treatment within a few days.

Investigations

The results of hemogram, total leukocyte count (TLC), differential leukocyte count (DLC), serum bilirubin, serum glutamic-oxaloacetic transaminase (SGOT), serum glutamic-pyruvic transaminase (SGPT), blood urea nitrogen (BUN), and serum creatinine levels for all patients are presented in Table 1. Serology for scrub typhus IgM was positive (by ELISA) in all four cases.

**Table 1:** Investigation results BUN: blood urea nitrogen; SGOT: serum glutamic-oxaloacetic transaminase; SGPT: serum glutamic-pyruvic transaminase

Parameters	Normal range	Case I	Case II	Case III	Case IV
Haemoglobin (gm/dl)	11-14	10.2	11.2	6.0	10.0
Total count(/mcl)	4000-11000	20500	8000	11000	7500
Differential count	P40-60%, L20-40%	P70	P75	P65	P73
Platelet count (/mcl)	150000-450000	130000	200000	180000	80000
Serum bilirubin (total mg/dl)	0.1-1.2	1.3	1.8	4.3	1.1
SGOT (iu/l)	5-40	53	82	250	48
SGPT (iu/l)	7-56	101	120	400	112
BUN (mg/dl)	7-20	17	13	15	19
Serum creatinine (mg/dl)	0.7-1.3	1.1	1.2	1.4	1.0

## Discussion

Severe scrub typhus infections can lead to severe consequences such as acute renal failure, myocarditis, hepatic dysfunction, and multi-organ involvement, with severity comparable to that of conditions causing ARDS. The complications typically begin to emerge following the initial week of the disease. Despite its general clinical features, scrub typhus is a significant and emerging illness, and it is often underdiagnosed. Scrub typhus can cause life-threatening disease, and hence management should start as soon as clinical suspicion indicates it and should be confirmed by serology later on.

The proliferation of *Orientia tsutsugamushi* in the endothelium of small blood vessels triggers cytokine release, which damages the endothelium, thereby leading to fluid leakage, platelet aggregation, polymorph and monocyte proliferation, and localized microinfarction. This ultimately causes peripheral gangrene and venous thrombosis. This process primarily affects several organs, such as the skin, kidneys, skeletal muscles, brain, lungs, and cardiac muscles [5]. Multiple organs can be infected with *Orientia tsutsugamushi* due to the lymphatic and blood systems. It primarily targets the liver's and spleen's macrophages [6]. Oxidative stress, a mechanism Rickettsia uses to damage endothelial cells and induce inflammation, results in both local and systemic vasculitis [7].

Despite the prevailing notion that scrub typhus causes diffuse endothelial infection, our case highlights the incidence of vasculitis leading to vaso-occlusion, a relatively uncommon occurrence. Systemic vasculitis-induced gangrene can have infectious or non-infectious origins, with infectious agents being the primary culprits. Examples include polyarteritis nodosa linked to HBV, and a small percentage of those with HCV contracting mixed cryoglobulinemia-related vasculitis. Other viruses capable of inducing vasculitis include cytomegalovirus, human T-cell lymphotropic virus (HTLV)-1, parvovirus B19, varicella-zoster virus, and HIV. Vasculitis can also be triggered by bacteria, fungi, or parasites, either by direct invasion of blood vessels or septic embolization. In addition to rickettsial diseases, syphilitic aortitis or cerebrovascular disease is another example of bacterially induced vasculitis.

Because the two types of etiologies of vasculitis require different therapeutic approaches, it is crucial to distinguish between them. Antimicrobial medications are used to treat bacterial, parasitic, and fungal infections. HBV infections leading to polyarteritis nodosa and HIV-related vasculitis can be successfully managed with a combination of antiviral medications and plasma exchanges. HCV-related cryoglobulinemic vasculitis is managed by antiviral medications and low-dose steroids [8]. Digital gangrene is a symptom associated with the development of systemic disorders and is often linked to smoking, high blood pressure, diabetes, obesity, and hyperlipidemia. Digital gangrene is a common symptom in conditions such as homocysteinemia, primary systemic vasculitis, and medium vessel vasculitis like polyarteritis nodosa. Rheumatoid arthritis and systemic lupus erythematosus (SLE) can also lead to premature atherosclerosis and gangrene. Thromboangiitis obliterans (TAO), commonly known as Buerger's disease [9], can result in claudication and digital gangrene.

The diagnosis of scrub typhus must be made by ruling out other infectious viral diseases, including dengue, infectious mononucleosis, HIV, and infectious bacterial diseases like typhoid, leptospirosis, and meningococcal disease, which can cause ARDS and vasculitis. In our case, we considered the possibility that scrub typhus could be the source of vasculitis because the ELISA serology for the disease was positive. Scrub typhus was consistent with the clinical features of the eight-year-old female patient (Case I), who presented with fever and gangrene and responded favorably to doxycycline treatment. The presence of positive scrub typhus serology further supports this diagnosis. In cases of multi-organ involvement associated with scrub typhus, we diligently ruled out all other possible differentials. Subsequently, pan-digital gangrene developed in the patient. We excluded other infectious and non-infectious conditions that can cause vasculitis, such as hepatitis B, hepatitis C, HIV, and SLE, leading us to conclude that the patient's gangrene was a result of scrub typhus vasculitis.

In light of an unusual manifestation of scrub typhus, we initiated a comprehensive evaluation of all patients with acute febrile illnesses who did not respond to treatment. While scrub typhus has been reported to manifest as acute cerebellitis in previous research by Didel et al. [10], in our Case II, it presented as meningoencephalitis with symptoms such as altered sensorium, fever, and convulsions. Patients with meningoencephalitis did not show improvement despite receiving definitive and supportive therapy. Following the favorable results of scrub serology, we initiated azithromycin (at 10 mg/kg per day single dose). In a study by Wilairatana et al. [11], a case of malaria and scrub typhus coinfection was reported, even though the patient did not present with fever or eschar markings. In one of our cases, however, the patient's condition did not improve despite implementing all necessary supportive measures, and no eschar mark was observed. Unexpectedly, the scrub serology test returned positive. Based on these findings, we concluded that scrub typhus should be considered as a potential diagnosis, even in the absence of an eschar mark.

In a research study conducted by Prakash et al. [12], acute kidney damage associated with scrub typhus was documented, and it was observed that the patient's condition improved with therapy. Conversely, in our Case IV, the patient showed signs of fever, rashes, and hematuria. Although a preliminary diagnosis of severe dengue was first entertained, dengue serology produced negative results, and the levels of antistreptolysin O titer (ASO) and C-reactive protein (CRP) were within normal ranges. The patient's blood pressure remained normal, and a urine analysis ruled out any additional renal reasons. Moreover, both infectious and non-infectious causes of fever were ruled out. Scrub typhus was identified as the likely culprit, especially given the patient's frequent travels to mountainous regions, which was later confirmed.

## Conclusions

Even though numerous illnesses can cause vasculitis, it is crucial to distinguish between infectious and non-infectious causes since these two require wholly distinct treatment approaches. Following an unexpected diagnosis of scrub typhus in a gangrene case, it was determined that scrub typhus should be investigated in all instances of acute febrile illness and multi-organ involvement, including CNS, renal, and hematological involvement, especially in patients with a history of visiting the mountainous areas of Kumaun in Uttarakhand. This conclusion was reached based on the rising occurrence of scrub typhus in the Kumaun region of northern India. Therefore, it is essential to conduct serological examinations for scrub typhus in all cases presenting with fever and multisystem involvement.
